# Prevalence, Severity and Impact of Foot Pain in 419 Pregnant Participants: The Queensland Family Cohort Study

**DOI:** 10.1002/jfa2.70132

**Published:** 2026-02-01

**Authors:** J. Raquel Fontes, Melinda M. Franettovich Smith, Felix Leung, Vicki L. Clifton, Julie Hides, M. Dilani Mendis

**Affiliations:** ^1^ School of Allied Health, Sport and Social Work Griffith University Nathan Queensland Australia; ^2^ Physiotherapy Department Fiona Stanley Fremantle Hospitals Group Perth Australia; ^3^ Physiotherapy The University of Queensland School of Health and Rehabilitation Sciences St Lucia Queensland Australia; ^4^ Mater Research Institute‐The University of Queensland Woolloongabba Queensland Australia; ^5^ Department of Physiotherapy Mater Adult Hospital Mater Misericordiae Limited South Brisbane Queensland Australia

**Keywords:** maternal health services, musculoskeletal pain, perinatal care, pregnancy, prevalence

## Abstract

**Background:**

Many hormonal, anatomical and biomechanical changes occur during pregnancy that may contribute to lower limb musculoskeletal dysfunction, including foot pain. Previous international studies have reported variable rates of foot pain in this population, but there is a lack of prospective longitudinal investigations. Improving the current understanding of foot pain is important to inform the development of management interventions during and after pregnancy. This study aimed to investigate the self‐reported prevalence, severity, frequency and impact of foot pain on work, activities and quality of life during pregnancy.

**Methods:**

Pregnant participants were recruited through the Queensland Family Cohort study conducted at a tertiary maternity hospital. Questionnaires were administered at enrolment (12–24 weeks' gestation) to collect demographics and at 24 weeks' gestation, 36 weeks' gestation and at the end of pregnancy (6 weeks postpartum) to measure the presence, severity and frequency of foot pain and symptoms and the impact of foot pain on work, activities and quality of life.

**Results:**

Four hundred and nineteen pregnant participants with a mean age of 32.2 (range 16–45) years and body mass index of 27 (range 17–52) were included. A high prevalence of foot pain was reported during pregnancy (44% up to 24 weeks; 56% up to 36 weeks, 54% up to the end of pregnancy). The severity of foot pain was mild to moderate and occasional in frequency. Foot pain had a mild to moderate impact on work, activities and quality of life during pregnancy. Participants with foot pain reported a lower perceived level of health during and at the end of pregnancy.

**Conclusion:**

Foot pain is a highly prevalent musculoskeletal problem that impacts work and quality of life during pregnancy. Pre‐natal and post‐natal care may provide an opportunity to assess and provide advice, treatment or appropriate referral for the management of foot pain.

## 1 Introduction

During pregnancy, the body undergoes hormonal and biomechanical changes known to contribute to musculoskeletal problems. While these are particularly well described in the literature for low back pain and pelvic girdle pain [1, 2, 3, 4], little is known about foot pain in this population. Previous studies report a prevalence of foot pain in pregnancy ranging from 31% to 42%, a figure considerably higher than that observed in nulliparous populations (22%) [5, 6, 7]. Studies investigating foot pain across each trimester have reported that the prevalence was greatest in the third trimester (37%–63%) compared to the second (17%–57%) and first (13%–47%) [8, 9, 10, 11]. For the first and third trimesters, foot pain was the second most common complaint (behind calf muscle cramps) [8]. This is notable as foot pain was more prevalent than the two musculoskeletal complaints most associated with pregnancy: low back pain and pelvic girdle pain. However, healthcare professionals rarely assess the foot health of expectant women as part of their routine care [12], suggesting that foot pain is a considerable problem that could be under‐recognised by clinicians.

Musculoskeletal pain during pregnancy is generally associated with a decline in perceived functional ability. Cross‐sectional surveys [9, 12, 13] revealed a deterioration in self‐reported foot health and mobility with a concomitant increase in functional and lifestyle limitations, suggesting that pregnancy leads to a marked decline in energy levels, physical activity and social capacity. However, little is known about the consequences of foot pain on women's overall function in work and daily activities, as well as their quality of life during pregnancy.

Improving the current understanding of foot pain will inform the development of management strategies targeted at women during each trimester of pregnancy. Therefore, the primary aim of this study was to investigate the self‐reported prevalence, severity, frequency and impact of foot pain on work, activities and quality of life during pregnancy. We hypothesised that the prevalence of foot pain would be high during pregnancy. We also hypothesised that foot pain would significantly impact work, daily activity and quality of life during pregnancy.

## 2 Methods

### 2.1 Setting

This study was conducted at the Mater Mothers' Hospital in Brisbane, Australia. The Mater Mothers' Hospital is a tertiary‐level obstetric hospital providing public and private maternity healthcare. The Mater Misericordiae Ltd Research Ethics Committee (HREC/MML/82387) and the Griffith University Human Ethics Committee (HREC/2022/211) granted ethics approval.

### 2.2 Participants and Data Collection

The Queensland Family Cohort (QFC) was a prospective, longitudinal cohort study following pregnant participants and their partners throughout pregnancy and postnatally. The QFC study and recruitment process have been previously described [14, 15]. For this study, a subset of participants who were 12–24 weeks pregnant and scheduled to give birth at the Mater Mothers' Hospital from 2018 to 2021 were recruited. Recruitment was conducted by a team of research midwives, nurses and research assistants, who contacted individuals face‐to‐face, via telephone or via messaging service. All participants were provided with an information statement and could withdraw from the study at any time. All participants provided written informed consent prior to participating. Individuals 12–24 weeks pregnant, of any age, background or risk category, who permanently resided in Queensland were invited to participate in the study. The goal was to obtain a sample representative of the Queensland population; therefore, all pregnant participants were included regardless of chronic or acute health conditions. Those who declined to participate or were unable to give informed consent were excluded from the study. As this study was an exploratory analysis nested within the larger QFC study, no specific power calculation for foot pain was undertaken.

Participants were recruited between 12 and 24 weeks of pregnancy and completed the online consent and enrolment survey at the time of recruitment, which included contact details, demographics, education, employment and medical history. Participants were then asked to complete a series of electronic questionnaires about their symptoms at 24 weeks' gestation (24W), 36 weeks' gestation (36W) and 6 weeks postpartum (6WPP), which were timepoints determined by the QFC study design. All surveys were completed online and delivered to participants via email at each time point using automatic calculations based on the reported estimated due date. All information collected for research purposes was de‐identified. Data were collected and managed using the Research Electronic Data Capture (REDCap) electronic data capture tools hosted at the University of Queensland [16, 17].

### 2.3 Foot Pain & Impact Measures

Participants were asked to indicate the severity and frequency of foot pain and its impact on work and activities during pregnancy. Responses for the severity of foot pain were presented on a 5‐point Likert scale from ‘none’ to ‘severe’. Responses for the frequency of foot pain were on a 5‐point Likert scale from ‘never’ to ‘always’. Responses for difficulties during work or activities and limitations to work due to foot pain were on a 5‐point Likert scale from ‘not at all’ to ‘extremely’ (Table 1). Due to the wording of the questions referring to the duration of pregnancy rather than the specific time point at which data was collected, the responses were interpreted as an ‘ever‐experienced prevalence’ up to that time point, rather than a strict point prevalence.

**TABLE 1:** Queensland family cohort study musculoskeletal function questionnaire at 24 weeks' gestation, 36 weeks' gestation and 6 weeks postpartum.

Section E.4 foot pain	
What level of foot pain have you had during pregnancy?	None Very mild Mild Moderate Severe
How often have you had foot pain during pregnancy?	Never Occasionally Many times Very often Always
Have your feet caused you to have difficulties in your work or activities during pregnancy?	Not at all Slightly Moderately Quite a bit Extremely
Were you limited in the kind of work you could do because of your feet during pregnancy?	Not at all Slightly Moderately Quite a bit Extremely

### 2.4 Quality of Life Measures

The EQ‐5D‐5L [18, 19] is a generic measure of health status consisting of a descriptive system questionnaire and a visual analogue scale. The descriptive system questionnaire assesses health in five dimensions (Mobility, Self‐Care, Usual Activities, Pain/Discomfort, Anxiety/Depression), each of which has five levels of response (no problems, slight problems, moderate problems, severe problems, extreme problems/unable to). The second part of the questionnaire consists of a visual analogue scale (EQ VAS) on which the participant rates their perceived health from 0 (the worst imaginable health) to 100 (the best imaginable health) [20]. Participants were asked to provide their responses based on how they would best describe their health ‘today’ [20]. The EQ‐5D‐5L measure was collected at 24W and 6WPP time points.

### 2.5 Statistical Analyses

All data were analysed using IBM SPSS Statistics (version 30, IBM Corp, Armonk, NY). Data were examined for outliers and normal distribution using summary statistics, histograms, normality plots and the Kolmogorov‐Smirnov test. Baseline characteristics of the participants were summarised using descriptive statistics. Continuous normally distributed variables were reported as mean and standard deviation, continuous non‐normal variables were reported as median and inter‐quartile range (IQR) and categorical variables were described as frequencies and percentages. All participants were categorised into two groups based on their response to the level of foot pain question: “No Foot Pain” (none) and ‘Foot Pain’ (very mild to severe) and their responses to questions regarding severity and frequency of foot pain and impact of foot pain on work and activities were summarised using descriptive statistics. For each dimension of the EQ‐5D‐5L descriptive system (Mobility, Self‐Care, Usual Activities, Pain/Discomfort, Anxiety/Depression), responses were dichotomised into two categories: ‘No problems’ (level 1) and ‘Any problems’ (levels 2, 3, 4 and 5). The frequency profile for each dimension of quality of life (No problems/Any problems) was reported for the No Foot Pain and Foot Pain groups. The visual analogue scale (EQ‐VAS), on which participants rated their perceived health from 0 (the worst imaginable health) to 100 (the best imaginable health), was reported for each foot pain group. The difference in the EQ‐VAS score between groups was assessed with a Mann‐Whitney *U* test (significance level *p* = 0.05). Missing data were deemed missing at random. An available case analysis or per‐time point missingness was conducted to maximise the sample size and avoid additional bias from excluding partial responders. Baseline characteristics of age and BMI at enrolment were compared for responders versus non‐responders at each time point (24W, 36W and 6WPP).

## 3 Results

### 3.1 Participant Demographics

Data from 419 pregnant participants were available for analysis. Participant demographics are presented in Table 2. The results for the available case analysis indicated no significant differences in age or BMI between responders and non‐responders (*p* > 0.05) at 24W and 6WPP, no difference in age between responders and non‐responders at 36W (*p* > 0.05), but there was a significant difference between groups at 36W for BMI at enrolment (*p* = 0.03) with responders having a lower BMI.

**TABLE 2:** Demographics of pregnant participants at enrolment in the Queensland family cohort study.

Demographics	*n* ^a^	Mean (SD)	Range (min–max)
Age (years)	413	32.2 (4.8)	16–45
Height (metres)	418	1.7 (0.1)	1.4–1.8
Usual pre‐pregnancy weight (kilogrammes)	418	66.5 (14.3)	39–130
Pregnancy weight at enrolment (kilogrammes)	402	72.0 (14.0)	43–140
BMI at enrolment	402	26.5 (5.1)	17–52
**Parity** **^b^**	259	**Frequency**	**Percentage (%)**
0		32	12.4
1		134	51.7
2		71	27.4
≥ 3		22	8.5

^a^ Number of participants are different due to missing data.

^b^ Number of previous pregnancies that have reached 20 weeks gestation.

### 3.2 Foot Pain & Impact Measures

**FIGURE 1:**
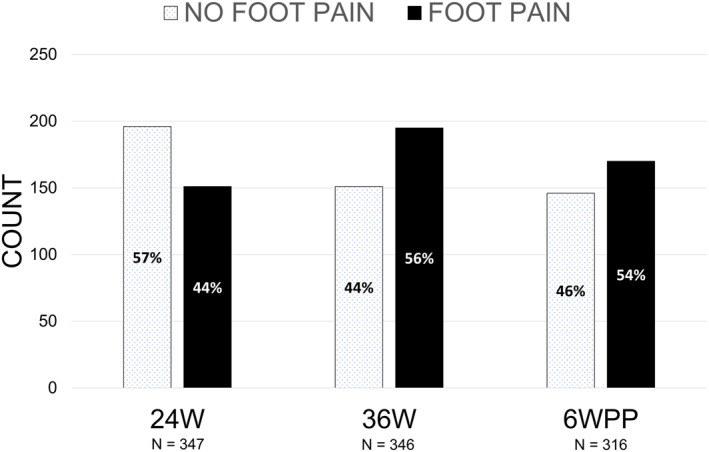
Prevalence of foot pain experienced during pregnancy. 24W, 24 weeks' gestation; 36W, 36 weeks' gestation; 6WPP, 6 weeks post‐partum; N, sample size.

A high prevalence of foot pain was observed during pregnancy (Figure 1). Up to 24 weeks of gestation, 151 of 347 (44%) participants had experienced foot pain during pregnancy. More than half of the participants (195 of 346, 56%) had experienced foot pain during pregnancy up to 36 weeks of gestation. By the end of the pregnancy, 170 of 316 (54%) participants had reported experiencing foot pain during pregnancy (Figure 1).

The severity of foot pain experienced by participants was ‘very mild’ to ‘mild’ during pregnancy (Table 3). The frequency of foot pain was occasional for 38%–42% participants during pregnancy and few experienced it many times (6%–12%), very often (1%–6%) or always (< 1%) (Table 3). Foot pain had a slight impact on work and activities for 16%–28% of participants, whereas for 3%–9% of participants, it had a moderate to severe impact on work and activities during pregnancy (Table 3). There was a limitation of work that could be performed by some of the participants (2%–16%) during pregnancy due to foot pain (Table 3).

**TABLE 3:** Questionnaire responses for severity and frequency of foot pain and its impact on work and activities.

	24W *n* (%)	36W *n* (%)	6WPP *n* (%)
Severity of foot pain	Total *n* = 347	Total *n* = 346	Total *n* = 316
None	196 (56.4)	151 (43.6)	146 (46.2)
Very mild	97 (28.0)	100 (28.9)	91 (28.8)
Mild	45 (13.0)	66 (19.1)	54 (17.1)
Moderate	9 (2.6)	29 (8.4)	23 (7.3)
Severe	0 (0.0)	0 (0.0)	2 (0.6)
Frequency of foot pain	Total *n* = 344	Total *n* = 342	Total *n* = 311
Never	189 (54.9)	148 (43.3)	135 (43.4)
Occasionally	131 (38.1)	143 (41.8)	119 (38.3)
Many times	21 (6.1)	33 (9.6)	37 (11.9)
Very often	3 (0.9)	16 (4.7)	20 (6.4)
Always	0 (0.0)	2 (0.6)	0 (0.0)
Difficulties with work or activities	Total *n* = 345	Total *n* = 345	Total *n* = 314
Not at all	276 (80.0)	217 (62.9)	214 (68.2)
Slightly	55 (15.9)	97 (28.1)	62 (19.8)
Moderately	10 (2.9)	21 (6.1)	29 (9.2)
Quite a bit	4 (1.2)	9 (2.6)	8 (2.5)
Extremely	0 (0.0)	1 (0.3)	1 (0.3)
Limitation of work	Total *n* = 346	Total *n* = 346	Total *n* = 314
Not at all	310 (89.6)	274 (79.2)	250 (79.6)
Slightly	28 (8.1)	55 (15.9)	44 (14.0)
Moderately	8 (2.3)	8 (2.3)	17 (5.4)
Quite a bit	0 (0.0)	7 (2.0)	2 (0.6)
Extremely	0 (0.0)	2 (0.6)	1 (0.3)

Abbreviations: 24W, 24 weeks' gestation; 36W, 36 weeks' gestation; 6WPP, 6 weeks postpartum; *n*, sample size; %, percentage.

### 3.3 Quality of Life Measures

EQ‐5D‐5L dimension responses are shown for participants with and without foot pain during and after pregnancy in Table 4. For participants with foot pain during pregnancy, 81% and 84% respectively had no problems with mobility, 93% and 95% had no problems with self‐care and 68% and 62% had no problems with usual activities, respectively. With regard to anxiety and depression, 59% and 55% of participants with foot pain had no problems during pregnancy, respectively. However, only 23% of participants with foot pain up to 24W reported no problems with pain/discomfort, whereas 78% experienced it. Following the end of pregnancy (6WPP), 55% of participants still had problems with pain/discomfort. The perceived level of health (EQ‐VAS) at 24 weeks' gestation for participants with foot pain was significantly lower (median 80; IQR 20; range 30–100; *n* = 149) compared with participants without foot pain (median 90; IQR 10; range 0–100; *n* = 192; *U* = 11,117; *z* = −3.58; *p* < 0.001). At 6 weeks postpartum, the perceived level of health (EQ‐VAS) for participants with foot pain was also significantly lower (median 80; IQR 20; range 8–100; *n* = 167) compared with participants without foot pain (median 85; IQR 15; range 30–100; *n* = 143; *U* = 9975; *z* = −2.53; *p* = 0.01).

**TABLE 4:** Distribution of EQ‐5D‐5L responses at 24 and 6 weeks post‐partum in the no foot pain and foot pain groups.

	No foot pain up to 24W	Foot pain up to 24W	No foot pain up to 6WPP	Foot pain up to 6WPP
EQ‐5D‐5L dimensions	*n* (%)^a^		*n* (%)^b^	
Mobility	*n* = 345		*n* = 306	
No problems	180 (92.8)	122 (80.8)	132 (93.0)	138 (84.1)
Any problems	14 (7.2)	29 (19.2)	10 (7.0)	26 (15.9)
Self‐care	*n* = 346		*n* = 312	
No problems	195 (100.0)	141 (93.4)	143 (98.6)	158 (94.6)
Any problems	0 (0.0)	10 (6.6)	2 (1.4)	9 (5.4)
Usual activities	*n* = 346		*n* = 312	
No problems	168 (86.2)	103 (68.2)	108 (74.5)	104 (62.3)
Any problems	27 (13.8)	48 (31.8)	37 (25.5)	63 (37.7)
Pain/Discomfort	*n* = 346		*n* = 312	
No problems	84 (43.1)	34 (22.5)	90 (62.1)	76 (45.5)
Any problems	111 (56.9)	117 (77.5)	55 (37.9)	91 (54.5)
Anxiety/depression	*n* = 346		*n* = 312	
No problems	132 (67.7)	89 (58.9)	97 (66.9)	92 (55.1)
Any problems	63 (32.3)	62 (41.1)	48 (33.1)	75 (44.9)

Abbreviations: 24W, 24 weeks' gestation; 6WPP, 6 weeks postpartum; *n*, sample size.

^a^ Percentage within the 24W No Foot Pain and Foot Pain groups.

^b^ Percentage within the 6WPP No Foot Pain and Foot Pain groups.

## 4 Discussion

A main finding of this study was the high prevalence of foot pain reported during pregnancy. Analysing data from 419 participants, this study found that 44%–56% of participants had foot pain during pregnancy. For most participants, the severity, frequency and impact of foot pain on work and activities were minimal during pregnancy; however, for some participants, foot pain was moderate‐severe and impacted their work and daily activities. Participants with foot pain during pregnancy up to 24 weeks of gestation had no problems with mobility, self‐care, usual activities and anxiety/depression. Nevertheless, pain/discomfort was a problem in early pregnancy and continued to be a problem 6 weeks after, with participants with foot pain reporting a lower perceived level of health.

We found a high prevalence of foot pain during pregnancy, comparable to that reported in previous studies [5, 6, 7, 8]. This prevalence is also similar to that presented in the literature on low back pain and pelvic girdle pain [21], which is usually reported to range from 45% to 56%. This may be due to adaptive changes in body biomechanics, such as increased plantar pressure, dynamic pronation and longer stance phase, which have been shown to peak in the last trimester of pregnancy [22]. In addition, a high weight‐to‐height ratio is a known risk factor for foot pain in the general population, leading to increased plantar pressure and pain intensity [23]. Similarly, in pregnant individuals, previous research has revealed that weight gain leads to increased plantar pressure and substantial changes in foot size and arch height, accounting for more than 90% of the variation in foot dimensions [24]. Consequently, the results of our study indicate that an appreciable number of women during the stages of pregnancy experience foot pain that could impact their work and daily activities.

Most respondents who experienced foot pain during pregnancy rated it as ‘very mild’ or ‘mild’ and felt that foot pain had a slight to moderate impact on work and daily activities, with minimal limitations to participants' physical capacity to meet the demands of their job. However, for some participants, foot pain was ‘moderate’ or ‘severe’ and caused a greater limitation to their work and activities. These results are supported by those of Lopez‐Lopez [25], who found that pregnant women presented lower scores on the dimensions of social capacity and vigour compared with nulliparous women. Our findings also partially corroborate those of Cassar & Formosa [12], whose study found that pregnant women experience a deterioration in mobility, lifestyle limitations and general health status throughout their gestation due to foot and ankle pain. In our study, the perceived level of health at 24 weeks of gestation and at 6 weeks postpartum for participants with foot pain during pregnancy was significantly lower than for participants without foot pain during pregnancy, but mobility and lifestyle were only minimally affected. Conflicting results may stem from several factors. Our study used the EQ‐5D‐5L to assess quality of life, whereas other tools, such as the Bristol Foot Score and the Foot Health Status Questionnaire, were translated into local languages and used elsewhere [26]. Second, different or unspecified time points were used in previous studies, which may have affected results. Thirdly, as our investigation was partially conducted during the COVID‐19 global pandemic, we cannot exclude the possibility that changes to participants' routine and activities due to intermittent local and regional lockdown measures may have affected results.

To our knowledge, this study is the first to investigate the prevalence of foot pain during pregnancy using a prospective longitudinal study design. The sample size, which has broad selection criteria, is also larger than many other studies examining the prevalence of foot pain during pregnancy.

The principal limitations of this study were due to the feasibility constraints imposed by the larger QFC study design [14, 15]. The broad scope of the whole study and the associated time commitments likely contributed to a decline in the number of participants who completed all questionnaires. The available case analysis indicated no differences in age or BMI between responders and non‐responders at 24W and 6WPP suggesting that data were likely missing at random. However, at the 36W timepoint, responders had a lower BMI at enrolment, suggesting a mild selection bias for the sample of participants surveyed at this timepoint. Secondly, the wording of the foot pain and impact questions could have been improved to better reflect the specific time periods investigated and avoid ambiguity. Because of the wording of the questions, the responses are best interpreted as an ‘ever‐experienced prevalence’ up to that time point, rather than a strict point prevalence. The higher numbers at later visits may therefore reflect the longer recall period, not only a true rise in new pain. Thirdly, this investigation was an exploratory study that aimed to provide an early insight into foot pain in pregnancy and its impact. It is acknowledged that musculoskeletal pain is complex in nature, with multiple contributing physiological and psychosocial factors. Accordingly, the pain/discomfort reported by the participants in the EQ‐5D‐5L may not necessarily be solely due to foot pain but could be caused by other factors during and after pregnancy. Fourthly, due to its voluntary nature, we cannot exclude the possibility of self‐selection bias in the recruitment process, as those with more severe symptoms might have more readily engaged with the research team. In addition, participants could not specify pre‐existing foot conditions, which part of the foot was affected and the nature of the pain (e.g., dull, sharp, throbbing, etc.). A body chart would have been a valuable addition to the questionnaire, providing a more comprehensive picture of participants' symptoms. Finally, this study offered several avenues to further investigate the nature of foot pain during pregnancy and its impact on maternal well‐being. Due to time constraints, we were unable to assess participants' activity levels during pregnancy. Further studies are warranted to determine whether sedentary behaviour, physical activity and exercise impact the prevalence of foot pain in this population.

### 4.1 Clinical Implications

Given the likely multifactorial nature of foot pain, optimal management of this condition may require a collaborative and multimodal approach. In this regard, allied health professionals such as podiatrists, physiotherapists and exercise physiologists could play a crucial role, by firstly assessing the foot health status of expectant women to identify those who would benefit from early intervention.

## 5 Conclusion

This study provides an essential first step in highlighting that foot pain may be a more prevalent problem during pregnancy than previously indicated. Although severity was mild and impact on work and activities was minimal, our study revealed that foot pain can impact quality of life during pregnancy. The findings from this investigation suggest that foot health and foot symptoms should be regularly assessed as part of antenatal and postnatal care to identify issues amenable to intervention or necessitating referral to other health professionals.

## Author Contributions

**J. Raquel Fontes:** data curation, formal analysis, visualization, writing – original draft, writing – review and editing. **Melinda M. Franettovich Smith:** conceptualization, methodology, formal analysis, writing – review and editing, supervision. **Felix Leung:** writing – original draft, writing – review and editing. **Vicki L. Clifton:** conceptualization, methodology, funding acquisition, project administration, resources, writing – review and editing, supervision. **Julie Hides:** conceptualization, methodology, writing – review and editing, supervision. **M. Dilani Mendis:** conceptualization, methodology, data curation, formal analysis, project administration, visualization, writing – original draft, writing – review and editing, supervision.

## Funding

The authors have nothing to report.

## Ethics Statement

Ethics approval was granted by the Mater Misericordiae Ltd Human Research Ethics Committee (HREC/MML/82387) and the Griffith University Human Ethics Committee (HREC/2022/211).

## Consent

All participants were provided with an information statement and could withdraw from the study at any time. All participants provided written informed consent prior to participating and gave consent for publication of de‐identified data.

## Conflicts of Interest

The authors declare no conflicts of interest.

## Data Availability

Access to research data may be obtained upon request to the corresponding author and consultation with the QFC governance committee and Mater Research.
